# McKillip Veterinary College

**Published:** 1895-07

**Authors:** 


### McKillip Veterinary College.

McKillip Veterinary College's initial year has been a very encouraging one to its projectors, and their announcement for the college year of 1895-96 betokens a confidence that is extremely gratifying. The severe entrance examination inaugurated will be rigidly maintained; the college fees have been raised from seventy-five to one hundred dollars, and a number of changes have been made in the Faculty, all of which tend materially to strengthen the school in many ways.

In conjunction with the college a bacteriological laboratory has been established for the manufacture of diphtheria antitoxin and several other blood-serum preparations. Dr. G. E. Krieger, the bacteriologist, will give in conjunction with the third-year course a special course of instruction in serum-therapeutics. A

course in the theory and practice of horseshoeing for shoeing-smiths has been established. Dr. J. H. Honan, M.D., D.V.S., graduate of the Rush Medical College, Chicago, and the American Veterinary College of New York City, has been elected to the Chair of Physiology. Dr. F. T. McMahon lectures on the subject of "Meat and Milk Inspection," and H. W. Wright, LL.D., on "The Laws of Warranty and Evidence."

A post-graduate course will be established after the first-year class has been graduated.

The degree to be conferred has not yet been decided upon, pending action of the Association of Faculties. Having only a junior class, no commencement exercises were held this year.

The McKillip Veterinary Medical Association, composed of the students, has already been established in conjunction with the school.

In the catalogue are printed the requirements for admission to the United States Veterinary Medical Association, a feature we think that might be well adapted by other schools.

